# Biological aspects of phage therapy versus antibiotics against *Salmonella enterica* serovar Typhimurium infection of chickens

**DOI:** 10.3389/fcimb.2022.941867

**Published:** 2022-08-04

**Authors:** Katarzyna Kosznik-Kwaśnicka, Magdalena Podlacha, Łukasz Grabowski, Małgorzata Stasiłojć, Alicja Nowak-Zaleska, Karolina Ciemińska, Zuzanna Cyske, Aleksandra Dydecka, Lidia Gaffke, Jagoda Mantej, Dorota Myślińska, Agnieszka Necel, Karolina Pierzynowska, Ewa Piotrowska, Edyta Radzanowska-Alenowicz, Estera Rintz, Krzysztof Sitko, Gracja Topka-Bielecka, Grzegorz Węgrzyn, Alicja Węgrzyn

**Affiliations:** ^1^ Laboratory of Phage Therapy, Institute of Biochemistry and Biophysics, Polish Academy of Sciences, Gdansk, Poland; ^2^ Department of Medical Microbiology, Faculty of Medicine, Medical University of Gdansk, Gdansk, Poland; ^3^ Department of Molecular Biology, Faculty of Biology, University of Gdansk, Gdansk, Poland; ^4^ Department of Cell Biology and Immunology, Intercollegiate Faculty of Biotechnology of University of Gdansk and Medical University of Gdansk, Gdansk, Poland; ^5^ Institute of Physical Culture, Kazimierz Wielki University, Bydgoszcz, Poland; ^6^ Department of Microbiology, Faculty of Biology, University of Gdansk, Gdansk, Poland; ^7^ Department of Animal and Human Physiology, Faculty of Biology, University of Gdansk, Gdansk, Poland

**Keywords:** phage therapy, *Salmonella enterica* infection, antibiotics, chicken, microbiome

## Abstract

Phage therapy is a promising alternative treatment of bacterial infections in human and animals. Nevertheless, despite the appearance of many bacterial strains resistant to antibiotics, these drugs still remain important therapeutics used in human and veterinary medicine. Although experimental phage therapy of infections caused by *Salmonella enterica* was described previously by many groups, those studies focused solely on effects caused by bacteriophages. Here, we compared the use of phage therapy (employing a cocktail composed of two previously isolated and characterized bacteriophages, vB_SenM-2 and vB_Sen-TO17) and antibiotics (enrofloxacin and colistin) in chickens infected experimentally with *S. enterica* serovar Typhimurium. We found that the efficacies of both types of therapies (i.e. the use of antibiotics and phage cocktail) were high and very similar to one another when the treatment was applied shortly (one day) after the infection. Under these conditions, *S.* Typhimurium was quickly eliminated from the gastrointestinal tract (GIT), to the amount not detectable by the used methods. However, later treatment (2 or 4 days after detection of *S.* Typhimurium in chicken feces) with the phage cocktail was significantly less effective. Bacteriophages remained in the GIT for up to 2-3 weeks, and then were absent in feces and cloaca swabs. Interestingly, both phages could be found in various organs of chickens though with a relatively low abundance. No development of resistance of *S.* Typhimurium to phages or antibiotics was detected during the experiment. Importantly, although antibiotics significantly changed the GIT microbiome of chickens in a long-term manner, analogous changes caused by phages were transient, and the microbiome normalized a few weeks after the treatment. In conclusion, phage therapy against *S.* Typhimurium infection in chickens appeared as effective as antibiotic therapy (with either enrofloxacin or colistin), and less invasive than the use the antibiotics as fewer changes in the microbiome were observed.

## Introduction

Infectious diseases of poultry are serious problems in the fields of veterinary medicine, human health, and the economy. Among bacterial pathogens infecting chickens and other species bred in aviculture, *Salmonella enterica* is one of the most important ones (Castro-Vargas et al., 2020). In humans, salmonellosis is diagnosed in almost 100 million cases every year, among which over 150 thousand are fatal. Salmonellosis is a foodborne disease, and infections of poultry are the major causes of the human illness as contaminated poultry produce serve as a way of pathogen transmission. Economic losses in farming and meat industry are also cause for concern as poultry infection with *Salmonella* results in meat disqualification from the market (Wernicki et al., 2017). The problem is even more pronounced in the light of the selection of strains of *S. enterica* resistant to many antibiotics (Castro-Vargas et al., 2020). Moreover, to limit the development of antibiotic resistance, the European Union have forbidden to use of these compounds in livestock feed, and a partial ban for this has been announced in the US (Wernicki et al., 2017). Therefore, finding novel therapies and preventive procedures against poultry infections caused by pathogenic bacteria, including *S. enterica*, became an urgent need (Ruvalcaba-Gómez et al., 2022).

One of the possible non-antibiotic therapies against pathogenic bacteria is the phage therapy, i.e. the use of phages, natural parasites or parasitoids of bacteria (Węgrzyn, 2022), to combat bacterial infections (Kortright et al., 2019). Apart from the potential applications of this kind of therapy in humans (Uyttebroek et al., 2022), many efforts were reported to use bacteriophages in preventive and therapeutic procedures in veterinary medicine (Gigante and Atterbury, 2019). In the latter case, an important factor is an economic value, and a specific economic evaluation of a potential phage therapy product for the control of infections caused by *S. enterica* in poultry has been performed, and valuable recommendations were presented (Torres-Acosta et al., 2019).

The attempts to employ phage therapy to treat *Salmonella*-infected chickens have been reported many times, starting over 30 years ago (Berchieri et al., 1991). The term ‘phage therapy and chicken’ gave in the PubMed database (https://pubmed.ncbi.nlm.nih.gov) over 100 records (as on May 7, 2022). A substantial part of these publications concerned the treatment of *S. enterica* infections, and the results in this field published to date have been summarized recently (Mosimann et al., 2021). The general conclusions made on the basis of that review of already performed studies were that the use of the phage therapy may lead to significant reduction of the number of living pathogenic bacteria in chickens and that profitable effects may be more pronounced in the short-term period and in older birds. The efficacy of the phage therapy concerned not only attempts to eliminate pathogenic bacteria, but also to normalize the gut microbiome, as demonstrated in recent reports (Upadhaya et al., 2021; Clavijo et al., 2022; Huang et al., 2022).

Despite quite a large body of data on the effects on the administration of bacteriophages in poultry (Mosimann et al., 2021), the vast majority of the reported studies on the phage therapy against *Salmonella* in chickens concerned solely effects of bacteriophages under various conditions. However, antibiotic therapy is still an important option in aviculture when bacterial infections occur. Therefore, the aim of this work was to compare the efficacy and various biological parameters between phage therapy and the use of antibiotics in chickens infected with *S. enterica* serovar Typhimurium which is one of the most common serovars responsible for poultry infections. The concept of this study was to use two recently discovered and described by us bacteriophages, vB_SenM-2 (Kosznik-Kwaśnicka et al., 2020a) and vB_Sen-TO17 (Kosznik-Kwaśnicka et al., 2020b), and two antibiotics, colistin and enrofloxacin, frequently used in veterinary practice to treat poultry although the latter antibiotic is quite controversial due to its adverse effects, but on the other hand it is one of the most effective antibacterial drugs used in veterinary medicine (it is not approved to be used in humans) (Sohail et al., 2021; Grabowski et al., 2022). The two phages were used together as a cocktail, as this combination of SenM-2 and vB_Sen-TO17 has been found recently to be effective against *S. enterica* strains and safe for eukaryotic cells in *in vitro* studies and in experiments with the *Galleria mellonella* animal model (Kosznik-Kwaśnicka et al., 2022). We tested the efficacy of both therapeutic procedures, using different times of treatment, the determined prevalence of *Salmonella* cells in chicken feces, estimated the ability of phages to penetrate various organs of chicken, tested the appearance of phage- and antibiotic-resistant bacteria, and evaluated microbiome composition in all tested groups of chickens. Thus, our study provides a complex view on the effects of the phage therapy in *Salmonella*-infected chickens (exemplified by the phage cocktail composed of vB_SenM-2 and vB_Sen-TO17) in comparison to antibiotic therapy with two drugs commonly used in veterinary medicine, colistin or enrofloxacin.

## Materials and methods

### Bacterial strain and bacteriophages

*S. enterica* serovar Typhimurium (strain no. 13) was obtained from The National *Salmonella* Centre at the Medical University of Gdansk (Gdansk, Poland) and was used and characterized in previous studies (Kosznik-Kwaśnicka et al., 2020a; Kosznik-Kwaśnicka et al., 2020b).

Phages vB_SenM-2 and vB_Sen-TO17 were described and characterized in our previous works (Kosznik-Kwaśnicka et al., 2020a; Kosznik-Kwaśnicka et al., 2020b) as was the efficacy of the mixture of those two phages on different laboratory models (Kosznik-Kwaśnicka et al., 2022)

### Culture media

Bacteria were grown in LB medium (Bio-Shop, Burlington, Canada) or on LB-agar plates. For minimal inhibitory concentration tests, Muller-Hinton broth (Graso Biotech) was used. For *S.* Typhimurium identification, Salmonella-Shigella agar, CHROMagar Salmonella PLUS and MSRV agars were used. All selective media used in this study were purchased from Graso Biotech (Starogard Gdański, Poland).

### Phage cocktail preparation

Phage lysates for the experimental cocktail were prepared in accordance to previously published protocols (Kosznik-Kwaśnicka et al., 2022). Briefly, bacterial host strain culture, grown overnight in LB medium, was added to fresh LB medium in a 1:100 ratio and incubated at 37°C with agitation at 150 rpm. At OD_600 =_ 0.15 (measured with SmartSpec PLUS, BIO-RAD, California, USA), the bacteria were infected with phages at a multiplicity of infection (m.o.i.) of 0.5 and incubated at 37°C until lysis occurred. For phage purification, polyethylene glycol 8000 (PEG8000) (BioShop, Burlington, Ontario, Canada) was added to a final concentration of 10% and stirred using a mixer (Carl Roth, Karlsruhe, Germany) overnight at 4°C. The precipitate was collected by centrifugation at 10,000 × *g* for 30 min, at 4°C (Avanti JXN-26, rotor JLA-8000, Beckman Coulter, Indianapolis, USA) and suspended in 0.89% NaCl (Alchem, Torun, Poland). PEG8000 was removed by adding 2 ml of chloroform (Alchem, Torun, Poland) and centrifugation at 4,000 × *g* for 15 min, at 4°C (Avanti JXN-26, rotor JS-13.1, Beckman Coulter, Indianapolis, USA). The procedure was repeated until no PEG8000 precipitate could be observed. Obtained lysates were then purified by centrifugation in sucrose (Sigma Aldrich, Saint Louis, Missouri, USA) gradients at 95,000 × *g* (Optima XPN-100, rotor SW32.1 Ti, Beckman Coulter, Indianapolis, USA) for 2.5 h (Green and Sambrook 2012). In order to remove the remaining sucrose, phage lysates were then dialyzed against 0.89% NaCl overnight at 4°C. The levels of endotoxin were checked using Purified Thermo Scientific™ Pierce™ LAL Chromogenic Endotoxin Quantitation Kit (no. 12117850, Thermo Fisher Scientific Inc., Paisley, UK) in accordance to the manufacturer’s protocol with some modifications (Kosznik-Kwaśnicka et al., 2022).

### Assessment of phage therapy efficacy in chickens

The assessment of phage therapy using *in vivo* chicken model was performed in the Pavilion of Experimental Birds Infections, University of Warmia and Mazury, Olsztyn, Poland (in accordance to the Local Ethics Committee for Experiments on Animals in Olsztyn permission no. 62/2019). The facility is equipped with a unique system of HEPA filters and appliances that maintains the pressure cascade in sanitary corridors, boxes and locks that exclude the possibility of contamination of the experimental rooms. Chickens (*Gallus gallus domesticus*) from each test group were grown in an 8 m^2^ common box.

Forced ventilation was used in the boxes (17 air changes per hour) and the air was cleaned with HEPA filters. The average humidity in which the birds were kept was 75% and the temperature was reduced from 33°C (first days of life) to 22°C (end of rearing), and the light cycle from 24 h light to 12/12 h light/dark at the light intensity of 10 lx. The full-fledged feed and water were provided in the libitum system (at will). Before the experiment, the meconium was collected from the birds and tested for *S. enterica* presence (ISO 6579-1:2017). Furthermore, the swabs from walls, doors, floors of the boxes and water and feed samples were collected and analyzed for the presence of *S. enterica* to demonstrate no such contamination. The preliminary experiment was then carried out where five chickens were infected with *S*. Typhimurium at the dose of 10^5^ PFU/ml, as described previously (Randall et al., 2005; Atterbury et al., 2007). After 5 days, the birds were sacrificed and the jejunum contents were then scanned for the presence of *S*. Typhimurium.

**Figure 1:**
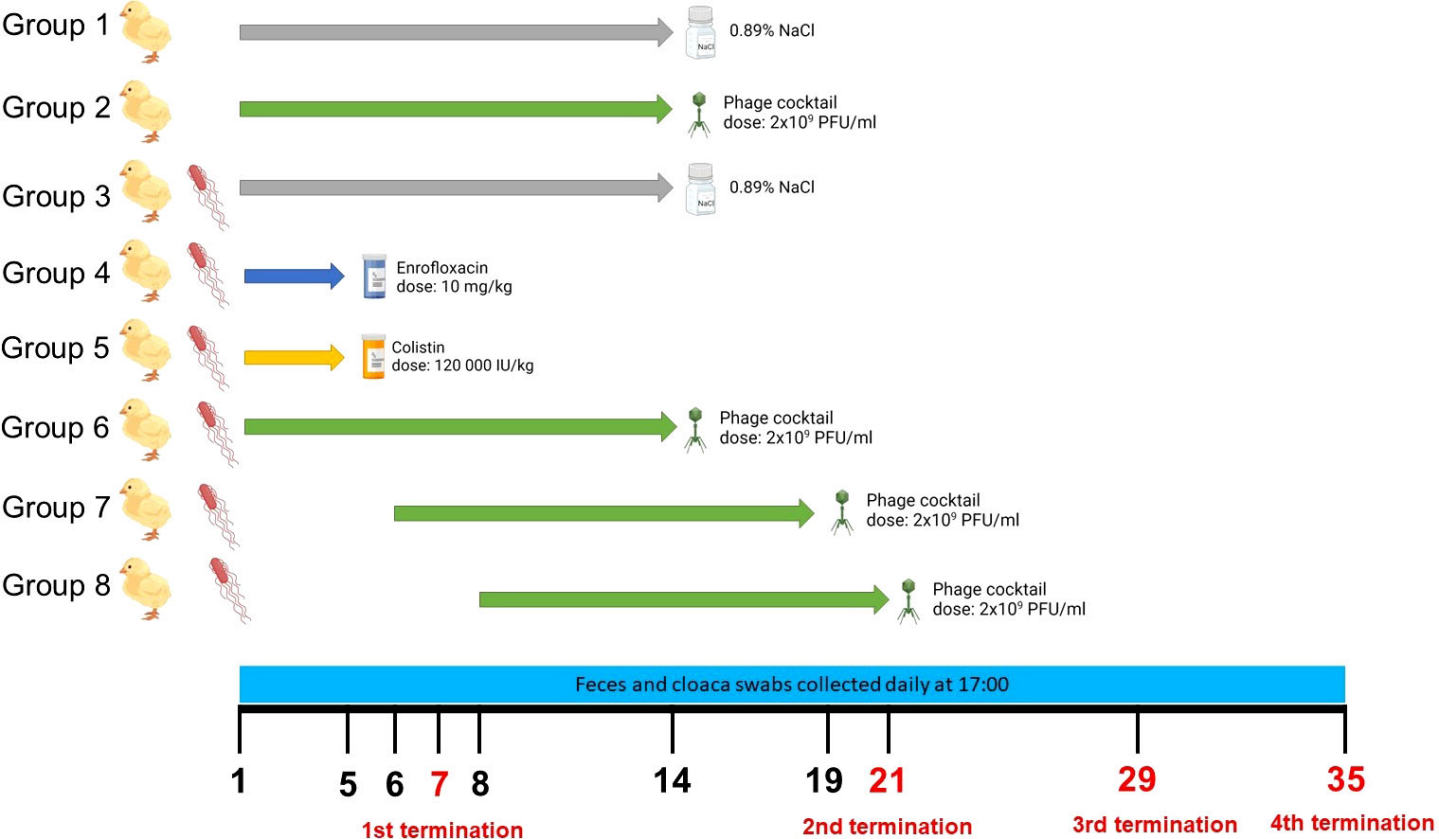
Experimental groups of chickens used in the experiments, the scheme of treatment variants of particular groups, and the time scale of sample collections and terminations. The figure was created with https://BioRender.com.

Seven days-old chickens were divided into 8 groups consisting of 25 individuals. On day 1 of the experiment, the birds from all groups, except groups 1 and 2, were infected with 1 ml of the suspension of *S.* Typhimurium (10^6^ CFU/ml) in 0.89% NaCl. On day 1 (24 h post infection), the treatment with phage cocktail (for 14 days; 1 ml of the phage suspension of 2 x 10^9^ PFU/ml (1 x 10^9^ PFU/ml of each phage) in 20 mM CaCO_3_ daily) in group 6, and antibiotic (for 5 days) in groups 4 (enrofloxacin; 10 mg/kg daily) and 5 (colistin; 120,000 IU/kg daily) began. Control groups (Groups 1 and 3) received 0.89% NaCl instead of the treatment. Group 7 received the first dose of the phage cocktail 2 days after the detection of *S.* Typhimurium in feces, and Group 8 – 4 days after *S.* Typhimurium detection. In both cases, the full cycle of phage therapy lasted 14 days. The detailed description of the times and dosages of therapeutics received by each group is presented in **Figure 1**. In order to monitor the presence of *S.* Typhimurium and phages, samples of chicken feces were collected daily. Additionally, cloaca swabs were collected from five randomly selected chickens. On day 7 of the experiment, after the end of antibiotic administration to chickens, 5 animals from each group were sacrificed and their organs were scanned for the presence of phages and bacteria. On day 21, after finishing the cycle of phage therapy, another 5 animals from each group were sacrificed. Two other terminations were made on days 28 and 35. Five ml of blood were collected from the animals before termination. For sterile blood collection, 5 ml heparinized syringes with a 25-gauge, 1-in-long needle were inserted into the brachial wing vein at a shallow angle (approximately 10–20°) andblood was collected into sodium heparin tubes. To obtain the plasma that was used in further analyses, whole blood was subjected to centrifugation (1,800 × g for 15 min at 4°C).

### *Salmonella* Typhimurium isolation from chicken feces and cloaca swabs

Screening for *S.* Typhimurium in chicken feces samples and cloaca swabs was done in accordance with the ISO 6579-1:2017 standards. The serotype of isolated bacteria was confirmed using serological identification with SIT EnTy Kit from Immunolab (Gdansk, Poland) in accordance with the manufacturer’s protocol. The level of *S.* Typhimurium in chicken fecal samples and cloaca swabs was analyzed. In brief, 0.5 g of feces sample were mixed with 5 ml of peptone water (Graso Biotech, Starogard Gdański, Poland) and incubated for 30 min at 37°C. After incubation, serial dilutions in peptone water were prepared and 50 µl of each dilution were spread onto CHROMagar Salmonella PLUS. The plates were then incubated at 37°C, overnight. After incubation, purple colonies were counted in order to calculate CFU/ml in chicken feces samples. For cloaca swabs, the samples were pooled and swabs were incubated for 30 min at 37°C with 5 ml of peptone water. After incubation, serial dilutions in peptone water were prepared and 50 µl of each dilution was spread onto CHROMagar Salmonella PLUS. The plates were then incubated at 37°C, overnight. After incubation, purple colonies were counted in order to calculate CFU/ml.

### Phage isolation from chicken feces and cloaca swabs

Two hundred µl of feces or swab sample were mixed with an equal volume of chloroform (Chempur, Piekary Śląskie, Poland) and centrifugated for 5 min, 3,000 × *g*, at room temperature in order to remove any bacteria present in the sample. One hundred µl of supernatant were then mixed with 200 µl of overnight *S*. Typhimurium culture and 4 ml of 0.7% top agar. The mixture was poured onto LB-agar plates. The plates were then incubated at 37°C, overnight. Afterwards, the plates were scanned for plaques and phage titer was counted.

### *Salmonella* Typhimurium isolation from chicken organs

After termination, chicken organs (brain, heart, stomach, liver, spleen, intestine, kidney and femoral muscle) were removed, cut with a sterile scalpel and then stamped on the Salmonella-Shigella agar by touching the agar surface with the organ’s cut surface, according to a previously described procedure (Shchebentovska et al., 2021). The stamps were then incubated at 37°C overnight, and after incubation they were scanned for the presence of bacterial colonies. If black colonies were detected, they would be transferred to LB-agar and later tested using SIT EnTy Kit in accordance with manufacturer’s protocol (however, no black colonies were detected in any tested samples). The sensitivity of this method was 1 cell per 1 cm^2^ of the sliced organ surface.

### Phage isolation from chicken organs

In order to isolate phages from chicken organs, samples of 0.5 g per organ were suspended in 1 ml of TM-buffer (50 mM Tris HCl, 10 mM MgSO_4_, pH 7.5) and homogenized using a manual tissue homogenizer. After homogenization, 100 µl of the organ sample were mixed with 200 µl of the overnight *S.* Typhimurium culture and 4 ml of Top Agar, and poured onto solidified LB-agar plates. The plates were then incubated at 37°C for 16 h and scanned for the presence of phage plaques afterwards. As the phages from the cocktail were identified using plaque morphology, the time of incubation was always the same and closely monitored. Since phages vB_SenM-2 and vB_Sen-TO17 have different plaque morphology, there was a possibility to distinguish one phage from another. The number of each plaque type was then counted separately and the PFU/g of each phage was calculated.

### Analysis of the development of resistance to phages and antibiotics during the therapy

Analysis of resistance to colistin and enrofloxacin was performed in accordance to EUCAST guidelines and previously described protocols (Andrews, 2001; Schön et al., 2019). In brief, solutions of colistin and enrofloxacin were diluted in Muller-Hinton broth and 100 µl of each dilution was transferred to a well of 96-well plate to obtain final concentrations of 10 µg/ml and 50 µg/ml, respectively. *S.* Typhimurium colonies that were spotted on CHROMagar and subsequently streaked onto fresh LB-agar medium and incubated overnight at 37°C. After incubation, bacteria were suspended in fresh Muller-Hinton broth to OD_600 =_ 0.01. One hundred µl of bacterial suspension were then added to an antibiotic solution and the plate was incubated at 100 rpm, 37°C, overnight. The absorbance was then measured at 600 nm at EnSpire Multimode Plate Reader (PerkinElmer, Inc. Waltham, Massachusetts, USA). The resistance threshold was established in accordance with EUCAST guidelines (Clinical breakpoints – bacteria v.9.0).

Phage resistance was tested using the spot test method. Double layered plates were prepared by pouring a mixture of 200 µl of overnight *S.* Typhimurium isolate culture and 4 ml top agar onto LB-agar plates and left to solidify. Serial dilutions of the phage cocktail were prepared, and then 2.5 µl of each dilution were spotted onto the plate. The droplets were then left to air dry and the samples were incubated at 37°C overnight. Phage titer (in PFU/ml) was then calculated and compared with the titer in the cocktail obtained with the control strain (*S.* Typhimurium 13).

The number of colonies used for the resistance test was based on *S.* Typhimurium CFU/ml from each day and calculated using online sample calculator (https://www.surveysystem.com/sscalc.htm)

### Phage plate neutralization test

The analysis was performed in accordance to protocols described by Łusiak-Szelakowska et al. (2014) and Letkiewicz et al. (2021). Briefly, chicken blood plasma was diluted from 1:10 to 1:1000. Fifty µl of the phage lysate (10^6^ PFU/ml) were added to 450 µl of each serum dilution. The control variant was performed by adding 50 µl of the phage lysate to 450 µl of LB-broth. The mixture was then incubated for 30 min at 37°C. After the incubation period, serial dilutions of each sample were prepared and titrated on double-layered agar plates. The plates were then incubated at 37°C overnight. The rate of phage inactivation was then calculated based on the formula:


$$K=2.3\times \left(\frac{D}{T}\right)\times \text{log}\left(\frac{P0}{Pt}\right)$$

where K is the inactivation rate, D is the reciprocal of the serum dilution, T is the time in minutes during which the reaction occurred (30 min), P0 is the phage titer at the start of the reaction (10^6^ PFU/ml), and Pt is the phage titer at time T=30.

The K value of less than 5 was considered to be a low level of phage inactivation, the K value between 5 and 18 was considered as a medium level of phage inactivation, and the K value above 18 was assessed as a high level of phage inactivation.

### Gastrointestinal microbiome analysis

Microbial genomic DNA was purified from gastrointestinal tract (GIT) content by using the PureLink Microbiome Purification Kit (Invitrogen, Carlsbad, CA, USA) according to the manufacturer’s manual. The DNA samples were sent to a commercial provider (Genomed S.A., Warsaw, Poland) for 16S rRNA gene PCR amplification, library preparation, Illumina MiSeq sequencing, and bioinformatic taxonomy analysis. The V3-V4 hypervariable region of the 16S rRNA gene was amplified using the primers 341F and 785R (Klindworth et al., 2013). The PCR reaction was conducted using Q5 Hot Start High0Fidelity 2 x Master Mix (NEB, Ipswich, MA, USA) according to the manufacturer’s instruction. Sequencing was performed on Illumina MiSeq using paired-end technology. An automatic initial analysis of data was conducted using the MiSeq device with the MiSeq Reporter v2.6 software. Bioinformatic analysis was performed using the QIIME software package (Caporaso et al., 2010) based on the reference sequence database GreenGenes v13_8 (DeSantis et al., 2006). To calculate the Shannon diversity index ( (Tucker et al., 2017)), which takes into account the abundance of each operational taxonomic unit (OTU), the PAST software version 4.09 (https://www.nhm.uio.no/english/research/infrastructure/past/) was used. The normality of the diversity index data was assessed using the Shapiro–Wilk test. Paired samples T-test was used to compare the diversity indexes with Group 1. The relative proportions of specific bacterial families in the animals groups studied were subjected to frequency analysis and then comparisons were made using the chi square test and IBM SPSS 21.0 software.

## Results

### Elimination of *Salmonella* cells after chicken infection and treatment with phages or antibiotic

To test the efficacy of elimination of *S.* Typhimurium from chickens, the birds were infected with this bacterium (at day 0 of the experiment) as described in Section 2.4. and depicted in **Figure 1**. Then, various treatments were applied. An antibiotic (enrofloxacin or colistin) treatment started one day after infection (denoted as day 1 of the experiment) and it was administered daily for 5 days. Treatment with bacteriophages (phage cocktail) started either one day after infection, or 2 or 4 days after detection of *S.* Typhimurium in chicken feces, and the phage cocktail was administered daily for 14 days. The efficacy of the treatment was assessed by measurement of the abundance of *S.* Typhimurium cells in feces and cloaca swabs.

**Figure 2:**
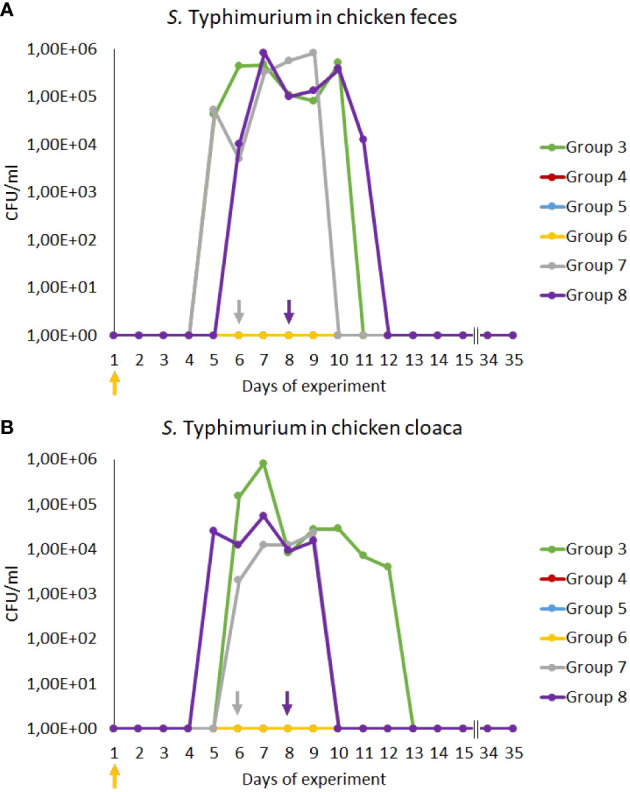
Prevalence of *Salmonella* Typhimurium 13 (CFU/ml) in chicken feces **(A)** and cloaca swabs **(B)** during the experiments. The days the groups began phage therapy are marked with arrows corresponding with colors to the group on the graph. No *S.* Typhimurium was isolated from samples from groups 4, 5 and 6, therefore, the lines overlap and are not visible on the graph.

We found that treatment with antibiotics (Groups 4 and 5) effectively eliminated *S*. Typhimurium as no cells of this bacterium could be detected during the whole experiment, contrary to infected chickens which were treated only with NaCl (Group 3) where 10^5^-10^6^ CFU/ml were present after 4-5 days post infection (**Figure 2**). A lack of *S. enterica* contamination was confirmed by the absence of this bacterium in feces and cloaca swabs of chickens uninfected with the *S.* Typhimurium 13 strain (Groups 1 and 2). Importantly, treatment with the phage cocktail which started one day post infection (Group 6) was as effective as antibiotic therapy, as no *S.* Typhimurium cells could be detected throughout the experiment (**Figure 2**). However, late initiation of the treatment with the phage cocktail, either 2 (Group 7) or 4 (Group 8) days after the detection of *S.* Typhimurium is feces resulted in drastically lower efficacy of elimination of this bacterium from the chicken gastrointestinal tract, as the cells of this bacterium were present for 4 days in feces and 2-4 days in cloaca swabs from the onset of administration of phages (**Figure 2**).

**Figure 3:**
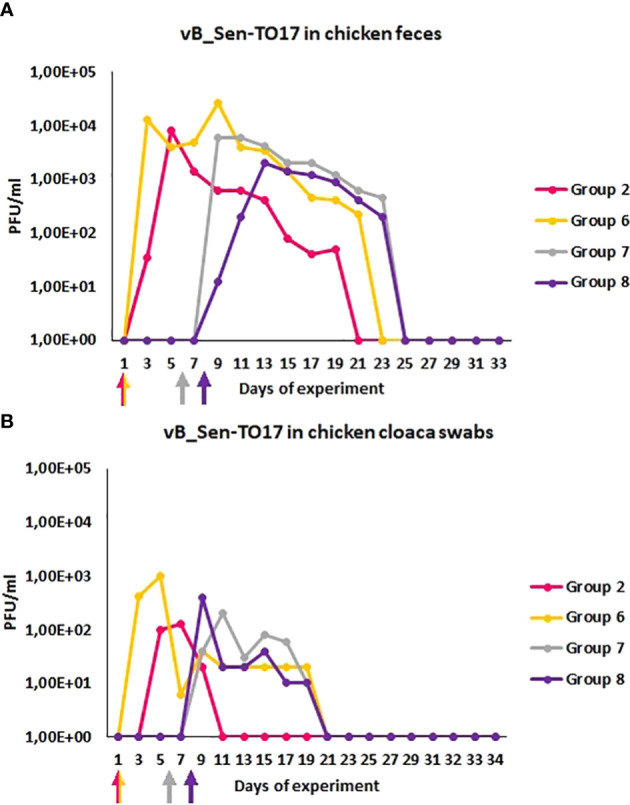
Prevalence of phage vB_Sen-TO17 (PFU/ml) in chicken feces **(A)** and cloaca swabs **(B)** during the experimental phage therapy. The days the groups began phage therapy are marked with arrows corresponding with colors of the group on the graph.

**Figure 4:**
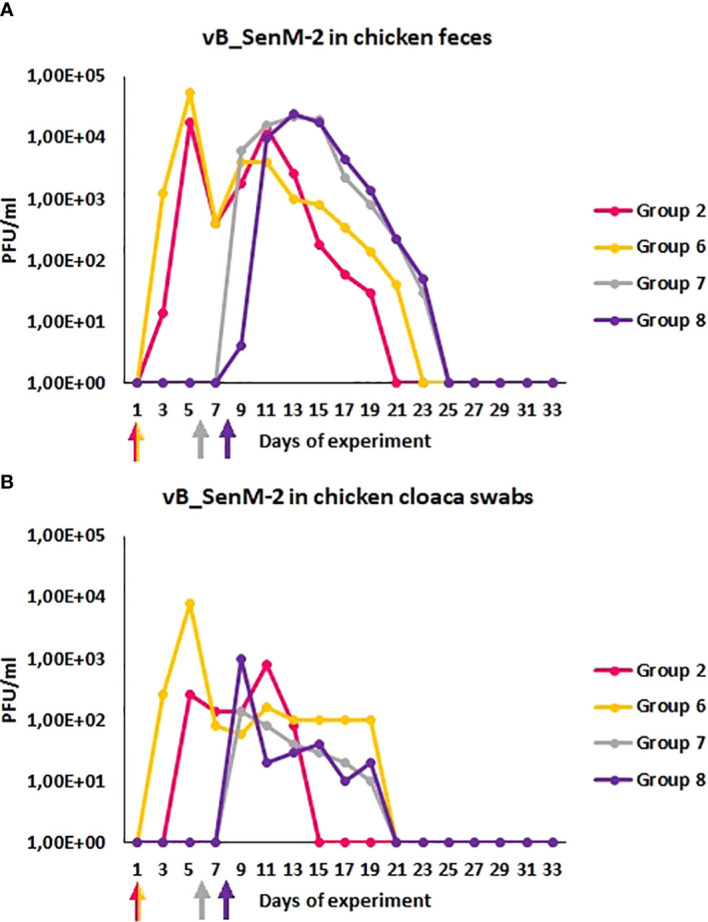
Prevalence of phage vB_SenM-2 (PFU/ml) in chicken feces **(A)** and cloaca swabs **(B)** during experimental phage therapy. The days the groups began phage therapy are marked with arrows corresponding with colors of the group on the graph.

**Figure 5:**
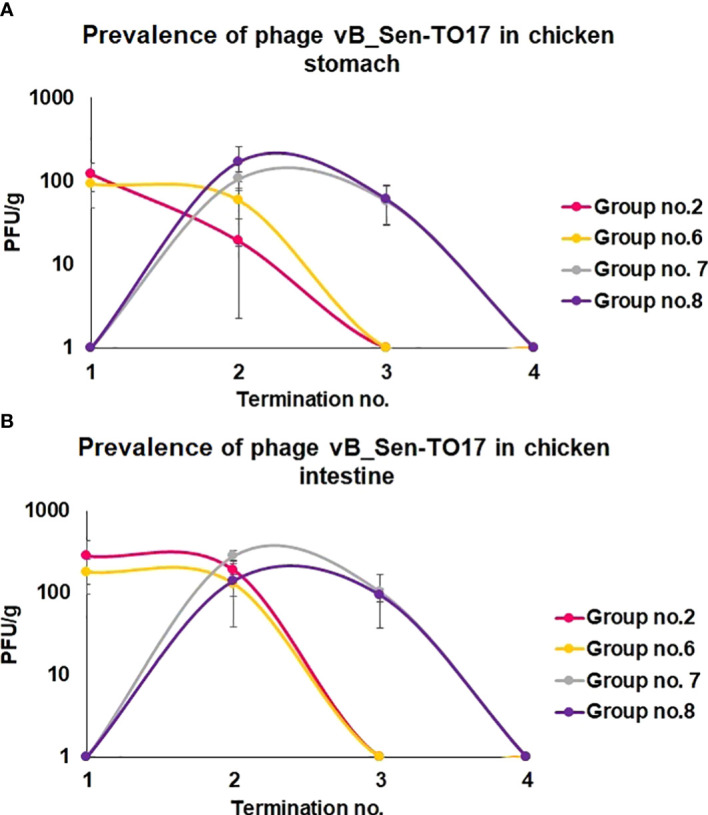
Changes in phage vB_Sen-TO17 titer (PFU/g) in chicken stomach **(A)** and intestine **(B)** during the experimental phage therapy at times of particular terminations.

**Figure 6:**
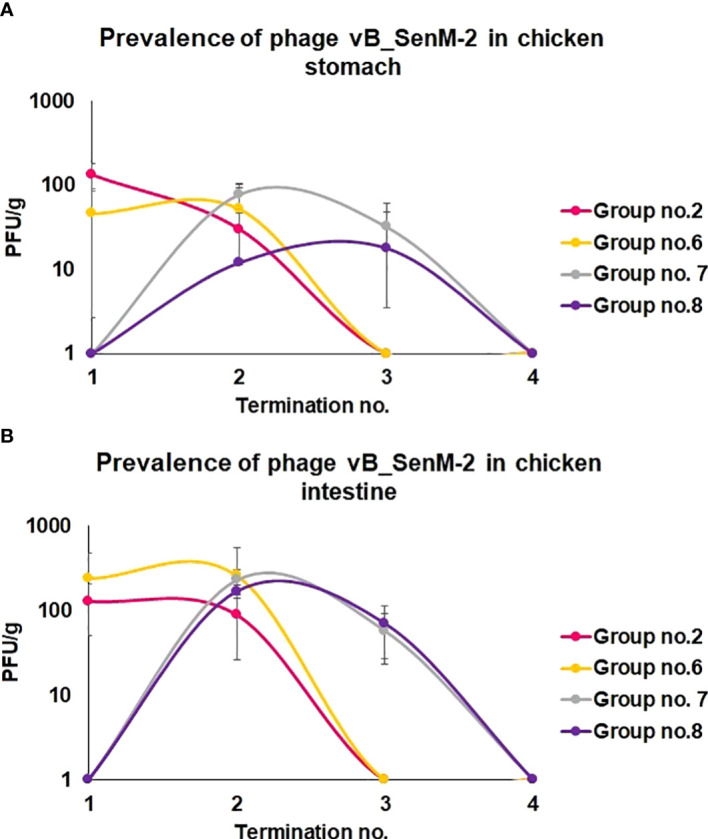
Changes in phage vB_SenM-2 titer (PFU/g) in chicken stomach **(A)** and intestine **(B)** during the experimental phage therapy at times of particular terminations.

We have also monitored the presence of phages vB_Sen-TO17 (**Figure 3**) and vB_SenM-2 (**Figure 4**) in feces and cloaca swabs of chickens. In the Groups which received bacteriophages (Groups 2, 6, 7, 8), both phages were present in both tested biological materials up to 25 days if they had a contact with their host, *S.* Typhimurium (Groups 6, 7, 8) while for about 1.5-2 weeks in the absence of the bacterium in which they might propagate (Group 2). Therefore, these bacteriophages may persist in the gastrointestinal tract of chicken for several days without the possibility of propagation, and for about 3 weeks if they can develop in their bacterial host, while they disappear from this habitat relatively shortly after the elimination of the susceptible bacteria (**Figures 3**, **4**). This conclusion was corroborated by experiments in which we have determined the presence of phages vB_Sen-TO17 (**Figure 5**) and vB_SenM-2 (**Figure 6**) in the chicken stomach and intestine after termination (terminations no. 1, 2, 3, and 4, were performed at days 6, 21, 28, and 35 of the experiment, respectively). Again, these phages could be detected for several days after eliminating their host from the environment (the lumen of the gastrointestinal tract).

### The presence of bacteriophages in different organs of chickens

**Figure 7:**
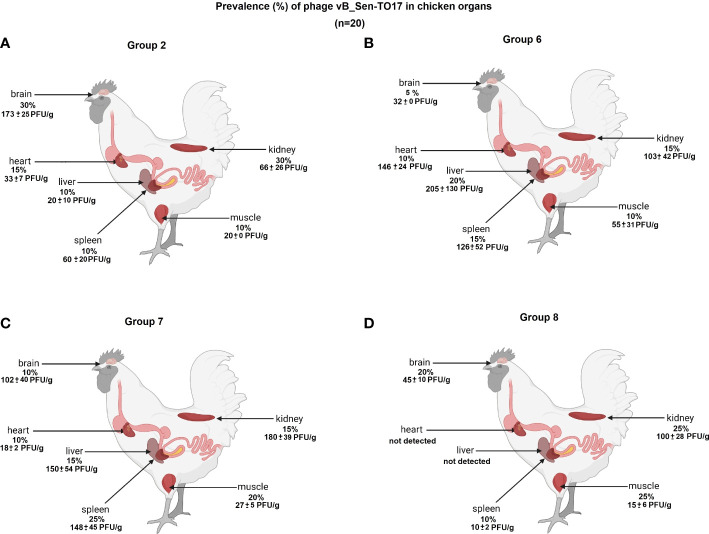
Presence of phage vB_Sen-TO17 (percent of chickens in which phages were detected is indicated; 100% = 25) in chicken organs in Groups 2 **(A)**, 6 **(B)**, 7 **(C)** and 8 **(D)**. The values represent mean phage titers (PFU/g) with SD.

**Figure 8:**
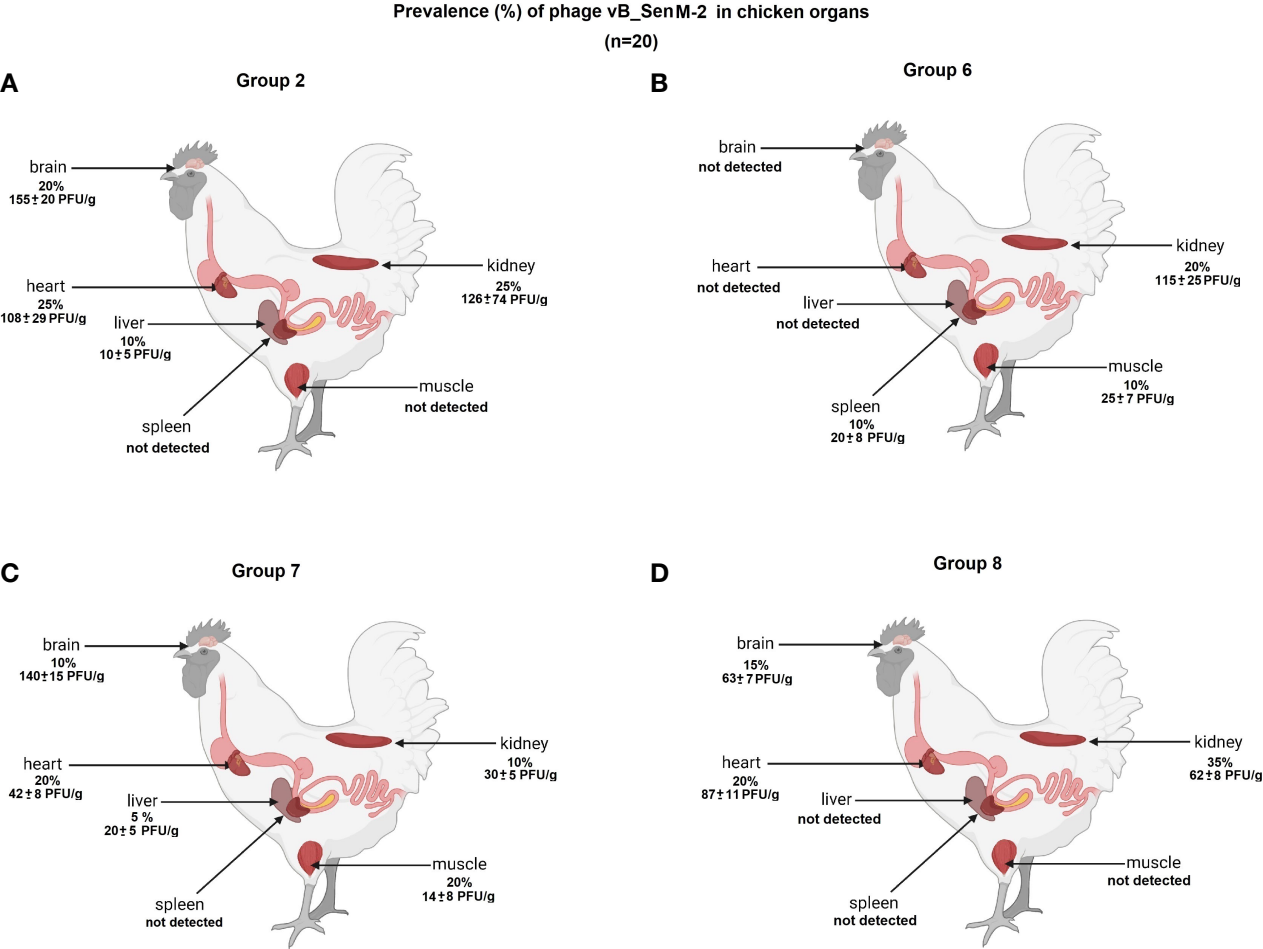
Presence of phage vB_SenM-2 (percent of chickens in which phages were detected is indicated; 100% = 25) in chicken organs in Groups 2 **(A)**, 6 **(B)**, 7 **(C)** and 8 **(D)**. The values represent mean phage titers (PFU/g) with SD.

We have investigated the distribution of orally administered bacteriophages in different organs of chickens. Following termination, the organs were tested for the presence of vB_Sen-TO17 (**Figure 7**; **Supplementary Table S1**) and vB_SenM-2 (**Figure 8**; **Supplementary Table S2**) phages. It is worth noting that the phages could be detected only in birds which were treated with the phage cocktail relatively shortly before the termination, i.e. at terminations no. 1 and 2 for Groups 2 and 6, and terminations no. 2 and 3 for Groups 7 and 8. Interestingly, orally administered bacteriophages could be detected in various organs, including the brain, heart, liver, spleen, muscle, and kidney. It is, however, worth noting that the phages were present in these organs in a relatively low percent of tested chickens; actually between 0 and 35% of birds contained bacteriophages in their organs.

The abundance of phages in the brain, heart, liver, spleen, muscle, and kidney was not high, and it reached maximally 1.8 x 10^2^ PFU/g. Interestingly, phage vB_Sen-TO17 (**Figure 7**) penetrated various organs considerably more efficient than vB_SenM-2 (**Figure 8**). This might be related to the sizes of both viruses, as vB_SenM-2 is significantly larger than vB_Sen-TO17 (compare Kosznik-Kwaśnicka et al., 2020a and Kosznik-Kwaśnicka et al., 2020b). It is worth noting that the highest titers of the latter phage were determined in the brain and kidney.

### Inactivation of bacteriophages by chicken plasma

We performed inactivation experiments in which we measured the efficiency of neutralization of bacteriophages by plasma isolated from chickens. Bacteriophage vB_SenM-2 was moderately neutralized by sera of chickens from Groups 2, 6, 7, 8, i.e. those which were treated with the phage cocktail (**Table 1**). A low level of the phage inactivation, corresponding to the nonspecific neutralization, was evident in experiments with sera from chickens which did not have a contact with vB_SenM-2 (**Table 1**). On the other hand, no high or moderate inactivation of phage vB_Sen-TO17 was observed by any serum, irrespective of the previous contact of chickens with this virus (**Table 2**). These results indicate that phage vB_SenM-2 is more immunogenic than vB_Sen-TO17.

**Table 1:** Inactivation levels of phage vB_SenM-2 in chicken sera, as measured by determination of the K value. Mean of values with SD are shown.

Experimental Group	Mean *K* value^a^		
	7 days of therapy	14 days of therapy	7 days after therapy
Group 1	1.91 ± 0.92	1.99 ± 0.48	1.16 ± 0.62
Group 2	6.13 ± 0.79	7.08 ± 1.71	7.21 ± 1.35
Group 3	1.07 ± 0.56	2.27 ± 0.85	1.14 ± 0.51
Group 4	2.14 ± 0.64	2.21 ± 0.66	2.98 ± 0.76
Group 5	2.44 ± 0.33	3.32 ± 0.43	2.63 ± 0.44
Group 6	6.46 ± 0.85	10.02 ± 0.91	10.10 ± 2.21
Group 7	5.67 ± 1.26	7.36 ± 1.28	6.51 ± 0.95
Group 8	7.41 ± 1.14	11.44 ± 2.36	11.27 ± 2.16

a Interpretation of the K values is as follows (according to Łusiak-Szelachowska et al., 2014): K ≤ 5, low inactivation; 5<K<18, medium inactivation; K≥18, high inactivation.

**Table 2:** Inactivation levels of phage vB_Sen-TO17 in chicken sera, as measured by determination of the K value. Mean of values with SD are shown.

Experimental Group	Mean *K* value^a^		
	7 days of therapy	14 days of therapy	7 days after therapy
Group 1	1.66 ± 0.63	1.56 ± 0.44	1.22 ± 0.32
Group 2	2.15 ± 0.39	2.10 ± 0.25	1.36 ± 0.22
Group 3	1.72 ± 0.37	1.85 ± 0.63	2.97 ± 0.48
Group 4	2.66 ± 0.47	2.98 ± 0.92	2.71 ± 0.71
Group 5	1.89 ± 0.59	1.74 ± 0.28	2.02 ± 0.62
Group 6	1.51 ± 0.66	2.17 ± 0.52	2.12 ± 0.77
Group 7	1.98 ± 0.64	2.14 ± 0.86	1.89 ± 0.75
Group 8	1.87 ± 0.81	2.09 ± 0.91	2.71 ± 0.82

a Interpretation of the K values is as follows (according to Łusiak-Szelachowska et al., 2014): K ≤ 5, low inactivation; 5<K<18, medium inactivation; K≥18, high inactivation.

### Assessment of antibiotic and phage resistance among surviving *S.* Typhimurium

We asked whether resistance to the investigated antibiotics and phages appeared among the survivors of *S.* Typhimurium found in chicken feces at various times of the experiment.

The survivors were tested for sensitivity to bacteriophages vB_SenM-2 and vB_Sen-TO17 (on the basis of titration of these phages on lawns of tested bacterial isolates), and MIC values for enrofloxacin and colistin were determined for these survivors. In each experiment, 100 isolates were investigated. We found that none of the survivors was resistant to either vB_SenM-2 or vB_Sen-TO17, and none of the survivors revealed significantly changed MIC values for both investigated antibiotics (which might indicate resistance to tested antimicrobial drugs). The summarized results of these experiments are presented in **Supplementary Tables S3–S6**, indicating no resistance to phages and antibiotics appeared during the experiment.

### Microbiome analysis

**Figure 9:**
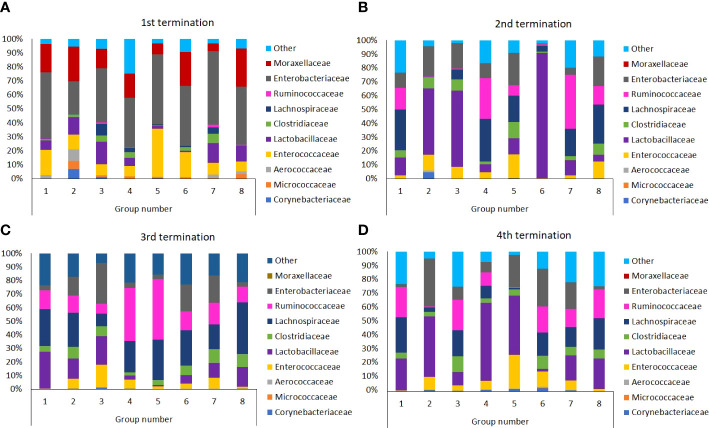
Differences in chicken intestinal microbiome among Groups from 1^st^ **(A)**, 2^nd^ **(B)**, 3^rd^ **(C)**, and 4^th^ **(D)** terminations.

As indicated in **Figure 9**, in young untreated chickens (Group 1, termination 1, 6^th^ day of the experiment; Shannon H index value: 1.495), Enterobacteriaceae was a predominant family, followed by Enterococcaceae and Moraxellaceae (χ2 = 102.374; p ≤ 0.001). Then, the microbiome composition was changed and then stabilized, thus, at terminations 2, 3, and 4, Lactobacillaceae and Lachnospiraceae predominated, with a significant contribution of Rumincoccaceae (χ2 = 84.112; p ≤ 0.01). Changes in GIT microbiomes of chickens treated with *S.* Typhimurium and/or antibiotics or phages were visible already at the time of the termination 1, however, they were significantly more pronounced later. Especially, treatment with *S.* Typhimurium alone (Group 3) resulted in a significant increase in the abundance of Enterobacteriaceae, as could be expected, but in addition, fraction of Enterococcaceae increased considerably (Shannon index value: 1.871). Treatment with antibiotics, either enrofloxacin (Group 4) or colistin (Group 5), caused a significant increase in the abundance of Enterococcaceae and Ruminococcaceae, followed by a predominance of Lactobacillaceae (χ2 = 124.105; p ≤ 0.001). Interestingly, early treatment of *S.* Typhimurium-infected chickens with the phage cocktail (Group 6) resulted in a huge predominance of Lactobacillaceae at the time of termination 2 (χ2 = 51.014; p ≤ 0.05). In this group, the microbiome normalized later (terminations 3 and 4), resembling that of the untreated chickens (Group 1). After initial changes in the microbiomes observed in chickens treated at later stages of the experiment with phages (Groups 7 and 8), which resembled those of birds which antibiotics were administered to (compare Groups 4 and 7, and 5 and 8 at the termination 2), the composition of bacteria also normalized at later times (terminations 3 and 4; Shannon index values: 1.955 and 1.633). This was in a strict contrast to chickens treated with antibiotics (Groups 4 and 5) where the microbiomes remained significantly different relative to the untreated birds (Group 1) even at the time of the last termination (fractions of Enterococcaceae, Lactobacteriaceae and Enterobacteriaceae remained significantly larger in chickens treated with enrofloxacin or colistin; (χ2 = 54.001; p ≤ 0.05). Interestingly, when the phage cocktail was applied to chickens not infected with *S.* Typhimurium (Group 2), the microbiome first resembled that in the GIT of *Salmonella*-infected birds (Group 3, see termination 2; Shannon index value: 1.373), then almost normalized (relative to untreated chickens, compare Groups 1 and 2 at termination 3; Shannon index values: 1.61 and 1.92 respectively), while finally Lactobacillaceae and Enterobacteriaceae predominated considerably (Group 2 at termination 4; (χ2 = 71.024; p ≤ 0.01).

**Figure 10:**
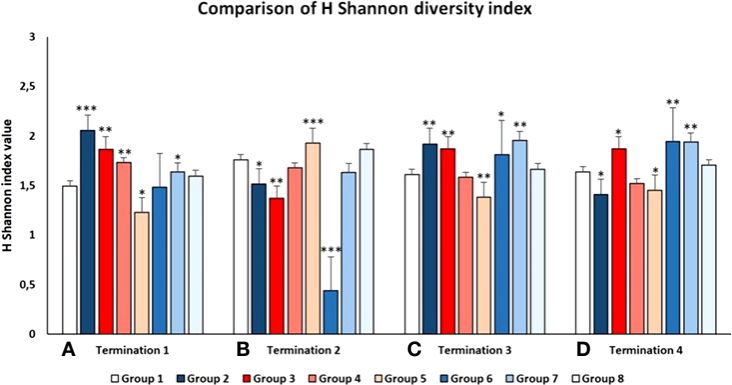
Shannon diversity index of the chicken intestinal microbiome in all tested Groups at 1^st^ **(A)**, 2^nd^ **(B)**, 3^rd^ **(C)**, and 4^th^ **(D)** terminations. Statistically significant changes relative to Group 1 are marked by asterisks, with * indicating p < 0.05, ** indicating p < 0.01, and *** indicating p < 0.001.

The above-described analyses were confirmed by calculating the Shannon diversity index that reflects how many different types (families of bacteria in this case) there are in a community (GIT microbiome in this case) (Tucker et al., 2017). The relative proportions of specific bacterial families in the animals groups studied were subjected to frequency analysis and then comparisons were made using the chi square test and IBM SPSS 21.0 software. As demonstrated in **Figure 10**, the diversity of bacterial families varied between groups and between terminations, generally in accordance with the analyses presented in the preceding paragraph. The most significant decrease in the Shannon index was observed in Group 6 (*S.* Typhimurium-infected chickens treated early with the phage cocktail) at the time of termination 2 (**Figure 10**), which strictly corroborates the results presented in **Figure 9**.

## Discussion

In the light of the massive appearance of antibiotic-resistant strains of *S. enetrica*, and introduced restrictions in the use of antibiotics in aviculture (Castro-Vargas et al., 2020), there is an urgent need to develop and introduce alternative methods for prevention and treatment of *Salmonella*-mediated infections of poultry. Phage therapy is one of the promising strategy in this field (Gigante and Atterbury, 2019). On the other hand, antibiotics are still important drugs used to combat infectious diseases in veterinary medicine, including poultry breeding (Deblais et al., 2020). Irrespective of many reports published previously on the use of bacteriophages to combat infections caused by *S. enterica* in poultry (summarized recently by Mosimann et al., 2021), those studies tested the effects of applications of phages to birds, without comparing them to those caused by antibiotics. Therefore, in this work, we compared biological aspects of phage therapy and the use of antibiotics in a large study on chickens infected experimentally with *S.* Typhimurium (**Figure 1**).

The major conclusion made on the basis of obtained results is that phage therapy is as effective as an antibiotic treatment in the elimination of *Salmonella* cells from the gastrointestinal tract of infected chickens if treatments are applied shortly after bacterial infection. This applies at least to the phage cocktail composed of two phages used in this study, vB_SenM-2 and vB_Sen-TO17 (characterized previously by us, see Kosznik-Kwaśnicka et al., 2020a, Kosznik-Kwaśnicka et al., 2020b), and two antibiotics, enrofloxacin and colistin, which are commonly used in veterinary medicine to treat poultry. Such a conclusion was based on the fact that when treatment with bacteriophages or antibiotics started no later than one day after oral infection with *S.* Typhimurium strain 13, no cells of this bacterium could be detected in feces and cloaca swabs starting from the next day after application of the therapeutic agents (**Figure 2**). However, when phages were administered later, at 2^nd^ or 4^th^ days after detection of *Salmonella* cells in chicken feces, then the elimination of the bacteria was significantly less efficient, and in fact, the results were comparable to those obtained in experiments with infected chickens which were not treated with any anti-bacterial agents. Therefore, it is worth to stress that to get positive results, phage therapy should be started as early as possible if *Salmonella*-mediated infection is detected. Otherwise the treatment might be of low efficacy if any. We suspect that since delayed phage treatment was applied after bacteria were able to already spread in the intestine, more time was needed for phages to effectively infect and lyse all present pathogenic bacteria (**Figure 2**). The growth conditions in the intestine are definitely different than those in laboratory, and phage propagation efficiencies also differ since they are strictly tied with life cycles of their hosts. Perhaps studying the lytic cycles and lysis profiles of bacteriophages in a laboratory under anaerobic or microaerophilic conditions could deliver more realistic data on how fast bacteriophages could clear out the already spread infection. However it is worth noting that after phage administration, the infection was eliminated after 3-4 days, regardless of the time of the administration (2 or 4 days after *Salmonella* was detected). This is also a promising result, since it shows that phages could be used for an ongoing infection and not just as a preventive measure. Given the fact that phages seem to have no negative influence on animals, perhaps the phage therapy might be proposed as a future way of treatment of bacterial infections without the need of flock termination.

An important feature is the time of persistence of the therapeutic agents in animal organisms. In the case of application of the phage cocktail, bacteriophages could be detected in the feces of chickens for 2-3 weeks, and then disappeared (**Figures 3**–**6**), most probably due to their disappearance (clearance) from the gastrointestinal tract in the absence of host bacteria in this environment. Interestingly, orally administered bacteriophages could be found in various organs of chickens, including the brain, heart, liver, spleen, muscle, and kidney (**Figures 7**, **8**). On the other hand, a number of virions detected in these organs was generally low, and never exceeded 200 phages per 1 g of the tissue, indicating that the penetration was of low efficiency.

The data of phage penetration to human and animal organs without the presence of pathogenic bacteria is a phenomenon that has been reported quite early (Murkerjee and Ghosh, 1962) and seems to be dependent on the type of phage used as well as the route of treatment administration. There are numerous studies reporting that phages are able to penetrate into organs of a mammal during therapy, especially into liver, spleen and kidney (Geier et al., 1973, Weber-Dąbrowska et al., 1987). Currently, the ability of phages to penetrate to bloodstream and into internal organs is highly researched topic (Dąbrowska et al., 2005; Dąbrowska and Abedon, 2019). The influence of this phenomenon on the therapy outcome is not yet clearly specified. However, to our knowledge, apart some theoretical suggestions (Tetz et al., 2017; Tetz and Tetz, 2018), there were no reports that phage penetration had negative effects to animals or humans, and it appears that phages are removed from the organism rather quickly once the administration is stopped (Dąbrowska and Abedon, 2019). This is also what we have observed during our studies, as once the cocktail was stopped to be administrated, the phages were cleared out from the organism after a few days and were not detected either in feces, swabs nor in internal organs (**Figures 3**–**6**).

An interesting question was whether chickens can produce anti-phage antibodies which are able to neutralize orally administered bacteriophages. We were not able to detect a considerable neutralization of the vB_Sen-TO17 phage by plasma from chickens (**Table 2**), however, some moderate neutralization of the vB_SenM-2 bacteriophage was detected by plasma of chickens which were treated with the phage cocktail (**Table 1**). These differences might be related to the fact that vB_SenM-2 is a significantly larger virus than vB_Sen-TO17 (Kosznik-Kwaśnicka et al., 2020a; Kosznik-Kwaśnicka et al., 2020b), thus, the former phage might be more immunogenic than the latter one. Importantly, no phage-resistant and antibiotic-resistant *Salmonella* cells could be detected in chicken feces after the experimental infection. This was true for both chickens untreated after infection and treated with antibiotics or phages. Therefore, the development of the resistance was not effective under the experimental conditions.

Gut microbiota plays a crucial role in the physiology of chickens and disturbances in the composition of the gut microbiome may cause serious disorders (Zhou et al., 2021), not only withing the gastrointestinal tract but also in other organs, including the central nervous system (Cao et al., 2021). In fact, although previous works on the use of phage therapy in chickens focused solely on testing the presence of certain pathogenic bacteria and/or specific symptoms in birds, recent studies indicated the importance of investigating microbiomes during and after specific treatment procedures (Clavijo et al., 2022; Lorenzo-Rebenaque et al., 2022). Therefore, we analyzed gastrointestinal (GIT) microbiomes of chickens from all tested groups, at times of each termination, i.e. days 6, 21, 28, and 35 of the experiment.

Our analyses indicated that both phages and antibiotics changed the composition of the chicken microbiome significantly. However, the changes caused by administration of the phage cocktail were transient, and the microbiome normalized during 2-3 weeks, while treatment with antibiotics (either enrofloxacin or colistin) resulted in a long-term (at least to the end of the experiment) disturbances in the composition of bacteria belonging to various families (**Figure 9**). Interestingly, the effects of bacteriophages on the microbiome were more pronounced when they were used without previous infection of chickens with *S.* Typhimurium than when the phage cocktail was applied after administration of the pathogenic bacteria. This effect might arise from the huge differences in the availability of sensitive host bacteria between both types of the experiments. When effective propagation was ensured in *Salmonella*-infected chickens, then effects of phages on other bacteria were minor. However, in the absence of the natural host, and administration of unnaturally high doses of bacteriophages, non-specific interactions and reactions might lead to more pronounced changes in the gastrointestinal microbiome. Calculation of the Shannon diversity index (**Figure 10**) corroborated these conclusions.

It is intriguing that in phage-treated chickens, in the absence of Salmonella, there were differences in intestinal microbiota. One might speculate that some secondary effects of non-specific interactions of phages with other bacterial species, as well as different abilities of the use of Salmonella-specific phages as nutrients by other bacteria, might result in temporary changes in the composition of microbiota. On the other hand, the changes were observed in the groups where Salmonella was also introduced, therefore, one cannot exclude the possibility that it was a combination of two factors that influenced the changes, namely the colonization by Salmonella and then its elimination that resulted in appearance of a niche that could be temporarily filled with other microorganisms. However, it was observed that after phage administration, the microbiome normalized, and the results were similar to those of the control groups.

In summary, phage therapy with the cocktail of vB_SenM-2 and vB_Sen-TO17 bacteriophages was as effective as the use of either enrofloxacin or colistin in elimination of experimentally provoked infection of chickens with *S.* Typhimurium. In both cases, the efficacy was high when the therapeutic agents were administered shortly after the infection. Phage therapy caused less pronounced changes in the gastrointestinal microbiome than the use of antibiotics which provides arguments for a higher safety of the former therapeutic approach in comparison to the latter one.

## Data availability statement

The datasets presented in this study can be found in online repositories. The names of the repository/repositories and accession number(s) can be found below: BioProject ID PRJNA838439.

## Ethics statement

The animal study was reviewed and approved by Local Ethics Committee for Experiments on Animals in Olsztyn (permission no. 62/2019, dated on July 30, 2019).

## Author contributions

KK-K prepared the phage lysates for cocktail creation, isolated the phages from feces, cloaca swabs and chicken organs, participated in sections of terminated chickens, performed the analysis of inactivation of phages by chicken blood plasma and the development of phage and antibiotic resistance, co-drafted the manuscript and was responsible for the visualisation of the results; MP helped plan and coordinate the experiment, participated and coordinated the sections of terminated chickens, prepared chicken blood plasma for further analysis, performed the statistical analysis for the analysis of microbiome diversity, ŁG assisted with phage lysates preparation and in phage isolation from feces and cloaca swabs, prepared chicken blood plasma and participated in sections of terminated chickens; MS isolated the DNA from feces and intestinal content for sequencing and participated in preliminary analysis of the results of microbiome diversity; AN-Z participated in isolation and identification of *S.* Typhimurium from chicken feces and organs; KC, ZC, AD, LG, JM, DM, AN, KP, EP, ER-A, ER, KS, and GT-B participated in sections of terminated chickens and isolation of organs for further analyses; GW participated in analyses of results and co-drafted the manuscript; AW was the principal investigator of the project, presented the concept of the study, planned the experiments, analysed data and co-drafted the manuscript. All authors revised and approved the final version of the manuscript.

## Funding

This work was supported by National Science Center (Poland) within project grant no. 2017/27/B/NZ9/00393.

## Acknowledgments

The authors thank the research team of the Pavilion of Experimental Birds Infections, University of Warmia and Mazury, Olsztyn, Poland, for their services during experiments with chickens. Students of the University of Gdansk who helped during the sections of chickens are thanked for their efforts.

## Conflict of interest

The authors declare that the research was conducted in the absence of any commercial or financial relationships that could be construed as a potential conflict of interest.

## Publisher’s note

All claims expressed in this article are solely those of the authors and do not necessarily represent those of their affiliated organizations, or those of the publisher, the editors and the reviewers. Any product that may be evaluated in this article, or claim that may be made by its manufacturer, is not guaranteed or endorsed by the publisher.

## References

[B1] Andrews J. M. (2001). Determination of minimum inhibitory concentrations. J. Antimicrob. Chemother. 48 Suppl 1, 5–16. doi: 10.1093/jac/48.suppl_1.5 11420333

[B2] Atterbury R. J. Van Bergen M. A. P. Ortiz F. Lovell M. A. Harris J. A. De Boer A.. (2007). Bacteriophage therapy to reduce salmonella colonization of broiler chickens. Appl. Environ. Microbiol. 73, 4543–4549. doi: 10.1128/AEM.00049-07 17526794 PMC1932804

[B3] Berchieri A. Lovell M. A. Barrow P. A. (1991). The activity in the chicken alimentary tract of bacteriophages lytic for salmonella typhimurium. Res. Microbiol. 142, 541–549. doi: 10.1016/0923-2508(91)90187-F 1947426

[B4] Cao C. Chowdhury V. S. Cline M. A. Gilbert E. R. (2021). The microbiota-Gut-Brain axis during heat stress in chickens: A review. Front. Physiol. 12. doi: 10.3389/fphys.2021.752265 PMC8563997 34744792

[B5] Caporaso J. G. Kuczynski J. Stombaugh J. Bittinger K. Bushman F. D. Costello E. K.. (2010). QIIME allows analysis of high-throughput community sequencing data. Nat. Methods 7, 335–336. doi: 10.1038/nmeth.f.303 20383131 PMC3156573

[B6] Castro-Vargas R. E. Herrera-Sánchez M. P. Rodríguez-Hernández R. Rondón-Barragán I. S. (2020). Antibiotic resistance in salmonella spp. isolated from poultry: A global overview. Vet. World 13, 2070–2084. doi: 10.14202/vetworld.2020.2070-2084 33281339 PMC7704309

[B7] Clavijo V. Morales T. Vives-Flores M. J. Reyes Muñoz A. (2022). The gut microbiota of chickens in a commercial farm treated with a salmonella phage cocktail. Sci. Rep. 12, 991. doi: 10.1038/s41598-021-04679-6 35046416 PMC8770602

[B8] Dąbrowska K. Abedon S. ,. T. (2019). Pharmacologically aware phage therapy: Pharmacodynamic and pharmacokinetic obstacles to phage antibacterial action in animal and human bodies. Microbiol. Mol. Biol. Rev. 83, e00012–e00019. doi: 10.1128/MMBR.00012-19 31666296 PMC6822990

[B9] Dąbrowska K. Switała-Jelen K. Opolski A. Weber-Dabrowska B. Gorski A. (2005). Bacteriophage penetration in vertebrates. J. Appl. Microbiol. 98, 7–13. doi: 10.1111/j.1365-2672.2004.02422.x 15610412

[B10] Deblais L. Kathayat D. Helmy Y. A. Closs G. Rajashekara G. (2020). Translating ‘big data’: better understanding of host-pathogen interactions to control bacterial foodborne pathogens in poultry. Anim. Health Res. Rev. 21, 15–35. doi: 10.1017/S1466252319000124 31907101

[B11] DeSantis T. Z. Hugenholtz P. Larsen N. Rojas M. Brodie E. L. Keller K.. (2006). Greengenes, a chimera-checked 16S rRNA gene database and workbench compatible with ARB. Appl. Environ. Microbiol. 72, 5069–5072. doi: 10.1128/AEM.03006-05 16820507 PMC1489311

[B12] Geier M. R. Trigg M. E. Merril C. R. (1973). Fate of bacteriophage lambda in non-immune germ-free mice. Nature 246, 221–223. doi: 10.1038/246221a0 4586796

[B13] Gigante A. Atterbury R. J. (2019). Veterinary use of bacteriophage therapy in intensively-reared livestock. Virol. J. 16, 155. doi: 10.1186/s12985-019-1260-3 31831017 PMC6909661

[B14] Grabowski Ł. Gaffke L. Pierzynowska K. Cyske Z. Choszcz M. Węgrzyn G.. (2022). Enrofloxacin–the ruthless killer of eukaryotic cells or the last hope in the fight against bacterial infections? Int. J. Mol. Sci. 23, 3648. doi: 10.3390/ijms23073648 35409007 PMC8998546

[B15] Green M. R. Sambrook J. Sambrook J. (2012). Molecular cloning: a laboratory manual. 4th ed (Cold Spring Harbor, N.Y: Cold Spring Harbor Laboratory Press).

[B16] Huang J. Liang L. Cui K. Li P. Hao G. Sun S. (2022). Salmonella phage CKT1 significantly relieves the body weight loss of chicks by normalizing the abnormal intestinal microbiome caused by hypervirulent salmonella pullorum. Poultry Sci. 101, 101668. doi: 10.1016/j.psj.2021.101668 35063807 PMC8784326

[B17] Klindworth A. Pruesse E. Schweer T. Peplies J. Quast C. Horn M.. (2013). Evaluation of general 16S ribosomal RNA gene PCR primers for classical and next-generation sequencing-based diversity studies. Nucleic Acids Res. 41, e1. doi: 10.1093/nar/gks808 22933715 PMC3592464

[B18] Kortright K. E. Chan B. K. Koff J. L. Turner P. E. (2019). Phage therapy: A renewed approach to combat antibiotic-resistant bacteria. Cell Host Microbe 25, 219–232. doi: 10.1016/j.chom.2019.01.014 30763536

[B19] Kosznik-Kwaśnicka K. Ciemińska K. Grabski M. Grabowski Ł. Górniak M. Jurczak-Kurek A.. (2020a). Characteristics of a series of three bacteriophages infecting salmonella enterica strains. Int. J. Mol. Sci. 21, 6152. doi: 10.3390/ijms21176152 PMC7503781 32858954

[B20] Kosznik-Kwaśnicka K. Grabowski Ł. Grabski M. Kaszubski M. Górniak M. Jurczak-Kurek A.. (2020b). Bacteriophages vB_Sen-TO17 and vB_Sen-E22, newly isolated viruses from chicken feces, specific for several salmonella enterica strains. Int. J. Mol. Sci. 21, 8821. doi: 10.3390/ijms21228821 PMC7700153 33233449

[B21] Kosznik-Kwaśnicka K. Stasiłojć M. Grabowski Ł. Zdrojewska K. Węgrzyn G. Węgrzyn A. (2022). Efficacy and safety of phage therapy against salmonella enterica serovars typhimurium and enteritidis estimated by using a battery of *in vitro* tests and the galleria mellonella animal model. Microbiolo Res. 261, 127052. doi: 10.1016/j.micres.2022.127052 35533436

[B22] Letkiewicz S. Łusiak-Szelachowska M. Międzybrodzki R. Żaczek M. Weber-Dąbrowska B. Górski A. (2021). Low immunogenicity of intravesical phage therapy for urogenitary tract infections. Antibiot (Basel) 10, 627. doi: 10.3390/antibiotics10060627 PMC8225094 34070276

[B23] Lorenzo-Rebenaque L. Malik D. J. Catalá-Gregori P. Marin C. Sevilla-Navarro S. (2022). Gastrointestinal dynamics of non-encapsulated and microencapsulated salmonella bacteriophages in broiler production. Animals 12, 144. doi: 10.3390/ani12020144 35049766 PMC8772543

[B24] Łusiak-Szelachowska M. Żaczek M. Weber-Dąbrowska B. Międzybrodzki R. Kłak M. Fortuna W.. (2014). Phage neutralization by sera of patients receiving phage therapy. Viral Immunol. 27, 295–304. doi: 10.1089/vim.2013.0128 24893003 PMC4076984

[B25] Mosimann S. Desiree K. Ebner P. (2021). Efficacy of phage therapy in poultry: A systematic review and meta-analysis. Poultry Sci. 100, 101472. doi: 10.1016/j.psj.2021.101472 34695636 PMC8554251

[B26] Mukerjee S. Ghosh S. N. (1962). Localization of cholera bacterio-phage after intravenous injection. Ann. Biochem. Exp. Med. 22, 73–76. 14477035

[B27] Randall L. P. Eaves D. J. Cooles S. W. Ricci V. Buckley A. Woodward M. J.. (2005). Fluoroquinolone treatment of experimental salmonella enterica serovar typhimurium DT104 infections in chickens selects for both gyrA mutations and changes in efflux pump gene expression. J. Antimicrobial Chemother 56, 297–306. doi: 10.1093/jac/dki189 15956100

[B28] Ruvalcaba-Gómez J. M. Villagrán Z. Valdez-Alarcón J. J. Martínez-Núñez M. Gomez-Godínez L. J. Ruesga-Gutiérrez E.. (2022). Non-antibiotics strategies to control salmonella infection in poultry. Animals 12, 102. doi: 10.3390/ani12010102 35011208 PMC8749512

[B29] Schön T. Matuschek E. Mohamed S. Utukuri M. Heysell S. Alffenaar J.-W.. (2019). Standards for MIC testing that apply to the majority of bacterial pathogens should also be enforced for mycobacterium tuberculosis complex. Clin. Microbiol. Infect. 25, 403–405. doi: 10.1016/j.cmi.2019.01.019 30771527 PMC7903878

[B30] Shchebentovska O. Kostynuk A. Zaika S. Kovalova L. Yevtukh L. Holubtsova M. (2021). Pathomorphological changes in the organs of chickens infected spontaneously by the species *Salmonella pullorum* on private farms in chernivtsi region. Regul. Mech. Biosyst. 12, 614–619. doi: 10.15421/022184

[B31] Sohail M. N. Rathnamma D. Priya S. C. Isloor S. Naryanaswamy H. D. Ruban S. W.. (2021). Salmonella from farm to table: Isolation, characterization, and antimicrobial resistance of salmonella from commercial broiler supply chain and its environment. BioMed. Res. Int. 2021, e3987111. doi: 10.1155/2021/3987111 PMC8514274 34660787

[B33] Tetz G. V. Ruggles K. V. Zhou H. Heguy A. Tsirigos A. Tetz V. (2017). Bacteriophages as potential new mammalian pathogens. Sci. Rep. 7, 7043. doi: 10.1038/s41598-017-07278-6 28765534 PMC5539208

[B32] Tetz G. Tetz V. (2018). Bacteriophages as new human viral pathogens. Microorganisms 6, 54. doi: 10.3390/microorganisms6020054 PMC6027513 29914145

[B34] Torres-Acosta M. A. Clavijo V. Vaglio C. González-Barrios A. F. Vives-Flórez M. J. Rito-Palomares M. (2019). Economic evaluation of the development of a phage therapy product for the control of salmonella in poultry. Biotechnol. Prog. 35, e2852. doi: 10.1002/btpr.2852 31131556

[B35] Tucker C. M. Cadotte M. W. Carvalho S. B. Davies T. J. Ferrier S. Fritz S. A.. (2017). A guide to phylogenetic metrics for conservation, community ecology and macroecology. Biol. Rev. 92, 698–715. doi: 10.1111/brv.12252 26785932 PMC5096690

[B36] Upadhaya S. D. Ahn J. M. Cho J. H. Kim J. Y. Kang D. K. Kim S. W.. (2021). Bacteriophage cocktail supplementation improves growth performance, gut microbiome and production traits in broiler chickens. J. Anim. Sci. Biotechnol. 12, 49. doi: 10.1186/s40104-021-00570-6 33858501 PMC8050931

[B37] Uyttebroek S. Chen B. Onsea J. Ruythooren F. Debaveye Y. Devolder D.. (2022). Safety and efficacy of phage therapy in difficult-to-treat infections: A systematic review. Lancet Infect. Dis. doi: 10.1016/S1473-3099(21)00612-5 35248167

[B39] Węgrzyn G. (2022). Should bacteriophages be classified as parasites or predators? Polish J. Microbiol. 71, 3–9. doi: 10.33073/pjm-2022-005 PMC9152906 35635166

[B38] Weber-Dabrowska B. Dabrowski M. Slopek S. (1987). Studies on bacteriophage penetration in patients subjected to phage therapy. Arch. Immunol. Ther. Exp. 35, 563–568. 3332066

[B40] Wernicki A. Nowaczek A. Urban-Chmiel R. (2017). Bacteriophage therapy to combat bacterial infections in poultry. Virol. J. 14, 179. doi: 10.1186/s12985-017-0849-7 28915819 PMC5602926

[B41] Zhou Q. Lan F. Li X. Yan W. Sun C. Li J.. (2021). The spatial and temporal characterization of gut microbiota in broilers. Front. Veterinary Sci. 8. doi: 10.3389/fvets.2021.712226 PMC8435590 34527716

