# Supplementary material for: Biological aspects of phage therapy versus antibiotics against Salmonella enterica serovar Typhimurium infection of chickens
Source: Front Cell Infect Microbiol. 2022 Aug 4;12:941867. doi: 10.3389/fcimb.2022.941867 (PMC9385949; doi:10.3389/fcimb.2022.941867)
Supplement: Supplementary file 1 [file DataSheet_1.pdf]

Supplementary Material

Table S1. Prevalence (%) of phage vB_Sen-TO17 in chicken internal organs (n=20), expressed also as mean values ±SD in PFU/g

Group no.	Prevalence of vB_Sen-TO17 in chicken internal organs											
	Brain		Heart		Liver		Spleen		Kidney		Muscle	
	%	PFU/g	%	PFU/g	%	PFU/g	%	PFU/g	%	PFU/g	%	PFU/g
2	30	173±25	15	33±7	10	20±10	10	60±20	30	66±26	10	20±0
6	5	32±0	10	146±24	20	205±130	15	126±52	15	103±42	10	55±31
7	10	102±40	10	18±2	15	150±54	25	148±45	15	180±39	20	27±5
8	20	45±10	0	0	0	0	10	10±2	25	100±28	25	15±6

Table S2. Prevalence (%) of phage vB_SenM-2 in chicken internal organs (n=20) expressed also as mean values ±SD in PFU/g

Group no.	Prevalence of vB_SenM-2 in chicken internal organs											
	Brain		Heart		Liver		Spleen		Kidney		Muscle	
	%	PFU/g	%	PFU/g	%	PFU/g	%	PFU/g	%	PFU/g	%	PFU/g
2	20	155±20	25	108±29	10	10±5	0	0	25	126±74	0	0
6	0	0	0	0	0	0	10	20±8	20	115±25	10	25±7
7	10	140±15	20	42±8	5	20±5	0	0	10	30±5	20	14±8
8	15	63±7	20	87±11	0	0	0	0	35	62±8	0	0

Table S3. Mean titer (±SD) of phage vB_Sen-M2 obtained on *S.* Typhimurium isolates from feces of chickens (control = 1 x 10^9 PFU/ml).

Mean titer of phage vB_Sen-M2 on *S.* Typhimurium isolates (PFU/ml)				
Group	Day of the experiment			
	5	7	9	11
3	7.52 ±2.55 x 10^8	2.07 ±1.08 x 10^9	9.01 ±3.16 x 10^8	7.43 ±5.88 x 10^8
7	8.77 ±2.13 x 10^8	7.83 ±1.25 x 10^8	1.16 ±4.22 x 10^9	-
8	7.93 ±3.26 x 10^8	6.44 ±2.41 x 10^8	5.36±3.16 x 10^8	7.68 ±3.66 x 10^8

Table S4. Mean titer (±SD) of phage vB_Sen-TO17 obtained on *S.* Typhimurium isolates from feces of chickens (control = 1 x 10^9 PFU/ml).

Mean titer of phage vB_Sen-TO17 on *S.* Typhimurium isolates (PFU/ml)				
Group	Day of the experiment			
	5	7	9	11
3	8.48 ±2.16 x 10^8	9.15 ±3.22 x 10^8	6.51 ±2.22 x 10^8	7.71 ±1.11 x 10^8
7	6.76 ±1.15 x 10^8	8.51 ±2.27 x 10^8	7.48 ±2.16 x 10^8	-
8	8.36 ±1.44 x 10^8	7.73 ±2.36 x 10^8	9.47 ±1.29 x 10^8	8.92 ±2.31 x 10^8

Table S5. MIC values of enrofloxacin with *S*. Typhimurium feces isolates (breakpoint MIC = 2 μg/ml)

MIC value of enrofloxacin of *S*. Typhimurium isolates (μg/ml)				
Group	Day of the experiment			
	5	7	9	11
3	0.06	0.06	0.06	0.06
7	0.06	0.06	0.06	-
8	0.06	0.06	0.06	0.06

Table S6. MIC value of colistin with *S*. Typhimurium feces isolates (breakpoint MIC = 2 μg/ml)

MIC value of colistin of *S*. Typhimurium isolates (μg/ml)				
Group	Day of experiment			
	5	7	9	11
3	0.5	0.5	0.5	0.5
7	0.5	0.5	1	-
8	0.5	1	0.5	0.5
